# Using Quantitative Masticatory Dysfunction to Inform Pain Management in Trigeminal Neuralgia Through Electromyographic Monitoring

**DOI:** 10.1111/jop.70035

**Published:** 2025-08-15

**Authors:** Alessia Riente, Alessio Abeltino, Cassandra Serantoni, Michele Maria De Giulio, Giada Bianchetti, Mariaconsiglia Santantonio, Giulio Cesare Passali, Stefano Capezzone, Rosita Esposito, Marco De Spirito, Giuseppe Maulucci

**Affiliations:** ^1^ Metabolic Intelligence Lab, Department of Neuroscience Università Cattolica del Sacro Cuore Rome Italy; ^2^ Department UOC Fisica per le Scienze Della Vita Fondazione Policlinico Universitario “A. Gemelli” IRCCS Rome Italy; ^3^ Complex Operational Unit of Otolaryngology Bambino Gesù Children's Hospital Roma Italy; ^4^ Complex Operational Unit of Ear, Nose and Throat Science Fondazione Policlinico Universitario “A. Gemelli” IRCCS Rome Italy; ^5^ Gruppo Fastal Blu Sistemi Rome Italy; ^6^ Digital Innovation Hub Roma Chirale S.r.l. Rome Italy

**Keywords:** chewing behavior, clustering analysis, electromyography, masticatory dysfunction, pain management, trigeminal neuralgia

## Abstract

**Background:**

Trigeminal neuralgia (TN) is a rare and debilitating condition characterized by severe, episodic facial pain, with an incidence of about five individuals per 100 000 annually, predominantly affecting women aged 50–70 years. TN is often difficult to diagnose; leading to underestimation or misdiagnosis and prolonged patient suffering.

**Objective:**

This study aimed to assess masticatory dysfunction in individuals with and without TN using an electromyographic device (“Chewing”) and evaluate its potential to quantify pain‐related dysfunction and inform treatment approaches.

**Methods:**

This observational study assessed masticatory dysfunction in TN patients and healthy controls using “Chewing” device. Masticatory behavior was monitored with apple and carrot as test foods, and parameters such as chewing time, number of chews, and chewing force were recorded. Participants were clustered based on masticatory patterns using an unsupervised learning approach.

**Results:**

Two distinct clusters of masticatory behavior emerged from the analysis. Cluster 1, representing 27.5% of TN1 patients, was characterized by prolonged chewing duration, a greater number of chewing cycles, and reduced chewing force compared to Cluster 0. Specifically, during apple mastication, Cluster 1 showed a 24% increase in chewing time (*p* = 0.02), a twofold increase in the number of chews (*p* < 0.001), and a 50% reduction in chewing force (*p* < 0.001). When chewing carrots, the number of chews increased by 57% (*p* < 0.001), while chewing force decreased by 64% (*p* < 0.001). Chewing frequency was also significantly higher in Cluster 1 for both food types (*p* < 0.001). Furthermore, a higher prevalence of TN1 patients was found in Cluster 1 compared to Cluster 0 (χ^2^ = 4.53, *p* = 0.05), suggesting an association between altered masticatory behavior and trigeminal neuralgia. Nonetheless, the presence of some TN1 patients in Cluster 0 indicates that masticatory function may remain intact in certain individuals, possibly due to milder pain symptoms or the development of compensatory coping strategies.

**Conclusions:**

“Chewing” device successfully quantified and differentiated masticatory patterns, providing valuable insights into functional adaptations. Subgrouping TN patients by masticatory behavior may guide personalized treatment strategies and improve patient outcomes.

## 1 Introduction

Classical trigeminal neuralgia (TN1) is a severe neurological condition affecting approximately five cases per 100,000 individuals annually [1], with the highest prevalence among those aged 50–70 [2]. Characterized by sudden, intense facial pain often triggered by common activities such as chewing, speaking, or washing the face, TN1 can significantly impair quality of life [3]. As the most common type of facial neuralgia, trigeminal neuralgia manifests through specific patterns related to the branches of the trigeminal nerve. The criteria set by the International Classification of Headache Disorders third edition (ICHD‐3) [4] for TN1 include experiencing repeated episodes of one‐sided facial pain that aligns with the trigeminal nerve area [5]. These pain episodes are brief, ranging from just a moment up to 2 min, and are described as intense, resembling an electric shock, and may feel like shooting, stabbing, or sharp [4, 6]. These pain attacks can be triggered by mild, everyday stimuli, like chewing, washing the face, or teeth brushing [7]. Masticatory activity not only serves as a potential trigger for pain episodes in TN1 patients, but also undergoes substantial adaptation as individuals attempt to cope with the condition. Neuroimaging and clinical studies have documented these adaptations: Zhang et al. [8] demonstrated, via MRI, the presence of muscle atrophy and edema in key masticatory muscles—such as the masseter, temporalis, and pterygoid—in patients with primary trigeminal neuralgia. Similarly, Mohammed et al. [9] observed structural changes in the muscles of mastication following stereotactic radiosurgery, suggesting both disease‐related degeneration and post‐treatment remodeling. Ichida et al. [10] further highlighted that sensory disturbances and impaired masticatory function can persist after therapeutic intervention, pointing to long‐term functional compromise. These adaptations can manifest as alterations in chewing patterns, with patients often adopting slower, less forceful chewing to mitigate discomfort. Over time, this can lead to decreased muscle function, changes in food intake, and even long‐term nutritional issues. Recent studies have demonstrated that TN1 patients exhibit measurable structural and functional changes in the masticatory muscles, such as atrophy and edema in the masseter, temporal, and pterygoid muscles, as revealed by magnetic resonance imaging (MRI) [11]. Several studies have concurrently identified the characteristics and functionality of masticatory muscles as a prognostic factor for various interventional strategies in the treatment of trigeminal neuralgia [9, 10, 12].

Based on this evidence, this study seeks to evaluate whether chewing behavior, as measured by an Electromyographic (EMG) device called “Chewing” [13], can be used as a tool to observe variations in masticatory function in TN1 patients. While chewing is only one of many triggers for trigeminal neuralgia, focusing on this aspect may offer objective data, especially when considering the potential adaptation of masticatory behavior in response to pain. This device could provide a quantitative assessment of dysfunction, allowing for objective analysis of behaviors directly related to pain adaptation, offering clinicians real‐time data for personalized treatment. Our aim is not to replace traditional diagnostic tools, such as the facial pain scale, but to complement them by providing a detailed analysis of mastication as a proxy for changes in pain management and treatment over time. Incorporating these assessments into treatment could allow for a more comprehensive approach to patient care, addressing not only acute pain but also longer‐term consequences on masticatory health and quality of life.

## 2 Materials and Methods

In this observational study, 96 participants were recruited to analyze chewing patterns using an EMG device known as “Chewing” considering two test foods (apple and carrot). Participants were enrolled voluntarily at the Università Cattolica del Sacro Cuore in Rome (Italy) in October 2023. This context enabled the recruitment of a heterogeneous population while ensuring adherence to ethical and methodological standards. All experimental procedures, including electromyographic recordings and data acquisition, were carried out in the laboratories of the Università Cattolica del Sacro Cuore, Department of Neuroscience, under controlled and standardized conditions. For further details on the characteristics of the participants, refer to Data S3.1. Prior to the experimental session, participants completed a comprehensive online questionnaire—covering demographics, health status, and medical history—prior to the experimental session. This data‐driven approach enabled the identification of key chewing metrics such as chewing time, number of chews, and chewing work. The study was conducted in accordance with the Declaration of Helsinki and approved by the Ethics Committee of Università Cattolica del Sacro Cuore (Protocol Code ID 3147). The study was conducted in accordance with the STROBE (Strengthening the Reporting of Observational Studies in Epidemiology) guidelines.

### 2.1 Exclusion and Inclusion Criteria

Participation in the study was open to individuals aged 18 years or older who volunteered. The cohort included both individuals with and without TN1. The presence of TN1 was self‐reported via a structured questionnaire and assessed in accordance with the diagnostic criteria established by the International Headache Society (IHS), aligning with the International Classification of Headache Disorders, 3rd edition (ICHD‐3) guidelines [4]. Only those participants who reported current symptoms consistent with these criteria were categorized as TN1 subjects. Healthy participants were those who did not report any history or symptoms consistent with TN1 and served as the control group in subsequent analyses. To ensure that variations in chewing patterns could be attributed specifically to TN1, several exclusion criteria were applied. Participants were excluded if they had active neoplastic disease in the head and neck area, a history of significant facial trauma affecting the facial skeleton (including the maxilla, zygomatic bones, nasal bones, and related structures), or had undergone major surgical procedures on the facial skeleton. Individuals who had received radiation therapy to the face or neck within the year before the assessment were also excluded. Additionally, participants with temporomandibular disorders (TMD), periodontal disease, or other chronic facial pain conditions were excluded to avoid confounding the results, as these conditions can independently affect masticatory function and facial pain. Older patients with complete removable dentures (both upper and lower arch) were excluded due to the known alterations in masticatory patterns associated with this type of prosthesis [14, 15]. Patients with diagnosed chewing disorders and noticeable bite abnormalities—such as crossbite, open bite, deep bite, or significant midline deviation—were excluded based on clinical assessment.

In addition, individuals who did not have a full set of natural teeth were excluded, as well as those with dental implants or other dental restorations that could significantly alter chewing patterns and masticatory force [16, 17]. A total of 136 individuals were screened for eligibility. After applying the inclusion and exclusion criteria, 96 participants were included in the final analysis. The detailed participant selection process is reported in the Figure S1.

### 2.2 Data Acquisition

The dataset analyzed includes 96 participants and 22 descriptive features, covering chewing patterns and additional information across three categories: anthropometric data (e.g., age, sex, and smoking habits), health and medical history (e.g., presence of neuralgia, lingual frenulum status, and dental treatments), and masticatory habits (e.g., nocturnal teeth wearing, prostheses use, preferred chewing side). For more details on the features considered, refer to Data S2.1.

### 2.3 Chewing Performance

**FIGURE 1:**
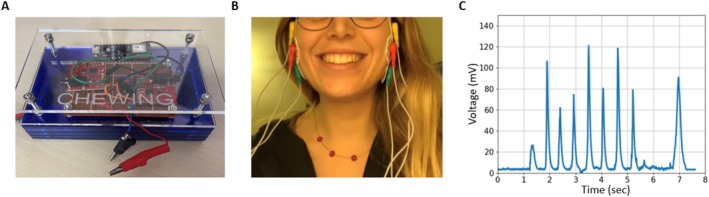
Chewing device and measurement. (A) The new prototype of the “Chewing” electromyographic device. (B) The measurement method is executed through the strategic placement of three electrodes on each of the subject's masseter muscles. One electrode is positioned at the center of the muscle to capture the primary muscle activity. Another electrode is placed at the terminal part of the muscle, ensuring comprehensive muscle engagement is recorded. The third electrode is affixed to the bony structure adjacent to the muscle, providing a reference point for the muscle's activity. (C) A sample EMG profile of masticatory activity. The profile captures the electrical signals generated by the muscles involved in chewing. When examining such a profile, one observes a dynamic and rhythmic pattern of muscle activity that corresponds to the various phases of the chewing process. Initially, as the masticatory process begins, there's a gradual increase in electrical activity. This uptick represents the engagement of muscles like the masseters and temporals, which are primary in the chewing action. The EMG signal shows a series of peaks and valleys, each corresponding to the closing and opening movements of the jaws. The amplitude of these signals increases as the muscles contract more forcefully to break down the food. The amplitude of the signals varies depending on the hardness and texture of the food being chewed. For tougher foods, the amplitude is higher, indicating stronger muscle contractions. The frequency of the peaks also aligns with the rhythm of the chewing cycle—faster chewing results in more frequent peaks.

The evaluation of masticatory behavior in this study is conducted using the “Chewing” device, an apparatus employing electromyographic technology [13, 18]. Upgrades include a rechargeable 11 V battery for extended lifespan and a more durable, user‐friendly plexiglass casing. Electrodes were strategically placed on the masseter muscles for accurate data capture, as illustrated in Figure 1A–C. The masseter was chosen for its accessibility and reliability, making the procedure more participant‐friendly.

The analysis considered six features of masticatory behavior: “chewing time (sec)”, “number of chews”, “cycle time (sec)”, “work (mV/s)”, “work rate (mV)”, and “frequency (1/s)”, calculated as the ratio of chews to time. For further details on the device and metrics, refer to Data S2.2.

### 2.4 Measurement Protocol

Participants completed an online questionnaire, provided informed consent, and confirmed understanding of the food ingredients to avoid allergic reactions. Two food samples, 30 g of “golden delicious” apple and carrot, were prepared to differ in hardness. Samples were sourced fresh, assessed for maturity using a refractometer (13°–14°Brix for apples [19]), and carefully portioned and stored to maintain quality. During testing, participants consumed the samples in order (apple first, then carrot) while seated comfortably, adhering to natural chewing patterns. Three electrodes were strategically placed on the masseter muscles to capture activity: one at the center, one at the terminal part, and one on a nearby bony structure for reference. For further details on the measurement protocol, refer to Data S2.3.

### 2.5 Analysis Pipeline

In this research, a case study was conducted to evaluate the chewing profiles of 96 participants using two distinct food samples, differentiated according to their hardness. Data analysis was performed through custom scripts written in Python (version 3.11), utilizing libraries such as scikit‐learn, scipy, and statsmodels. The sample size was based on the available data. This involved the selection of specific masticatory characteristics (“chewing time (sec)—apple”, “number of chews—apple”, “chewing time (sec)—carrot”, “number of chews–carrot”, and “work (mV/s)–carrot”; for more details, refer to Data S2.4), followed by the application of the *t*‐Distributed Stochastic Neighbor Embedding (t‐SNE) algorithm (“TSNE” package from the “sklearn.manifold” library). This algorithm facilitated an unsupervised clustering using the K‐Means method (“KMeans” package from the “sklearn.cluster” library), which successfully divided data into two distinct groups (labeled 0 and 1), based on achieving the optimal silhouette score (“silhouette_score” package from the “sklearn.metrics” library). Following this clustering, a comprehensive statistical analysis was conducted. This analysis encompassed the use of the *T*‐test and the Mann–Whitney test for evaluating continuous variables, alongside the Chi‐square test for categorical variables (“shapiro, mannwhitneyu, ttest_ind, chi2_contingency” package from the “scipy.stats” library). The threshold for statistical significance was established at a *p*‐value of 0.05. FDR correction was applied to the obtained *p*‐values (“multipletests” package from the “statsmodels.stats.multitest” library). Subsequently, a statistical power calculation was performed using the “TTestIndPower” package from the “statsmodels.stats.power” library, based on the final sample size (*n* = 96), and yielded a power value of 0.75. This indicates an adequate level of power to detect effects in this type of analysis, suggesting that the study is sufficiently powered to identify significant differences. The decision not to directly compare patients with TN versus those without TN was based on the exploratory nature of this study. Our primary goal was to investigate whether chewing patterns alone, independent of predefined clinical labels, could reveal distinct groupings related to the presence of trigeminal neuralgia (TN). This led us to choose an unsupervised clustering approach, allowing the data to reveal natural divisions in chewing behavior without the bias of categorizing participants a priori based solely on their TN status. By performing this type of cluster analysis, we sought to determine if there were identifiable patterns in mastication that could serve as potential indicators of TN, which may not have been apparent through direct comparison alone. This method enabled us to explore the possibility of subtle variations within the groups that may otherwise be missed when using a strictly binary classification of TN versus non‐TN patients.

Furthermore, this approach helps to identify potential subgroups of patients who, despite not being clinically diagnosed with TN, may exhibit chewing patterns similar to those of TN patients.

For further details on the algorithms and methods employed, please refer to Data S2.5–S2.7.

## 3 Results

A total of 96 participants who fulfilled the inclusion criteria completed the study protocol and were included in the analysis. No participants were excluded after enrollment.

### 3.1 t‐SNE‐Based Cluster Analysis and Silhouette Scoring

**FIGURE 2:**
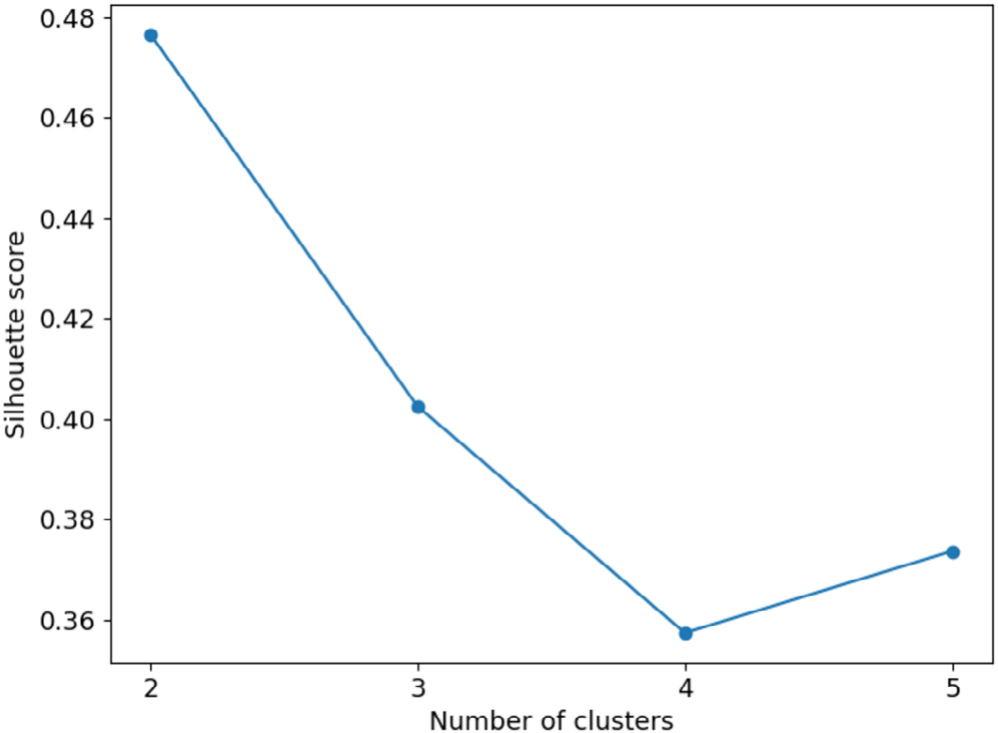
Variation of the silhouette score with different cluster counts. This graph, created with a python code, illustrates how the Silhouette score, a measure of clustering effectiveness, changes as the number of clusters (*k*) in the K‐Means algorithm is altered. It provides a visual representation of the score's trend, highlighting the optimal cluster count that maximizes clustering quality within the dataset.

**FIGURE 3:**
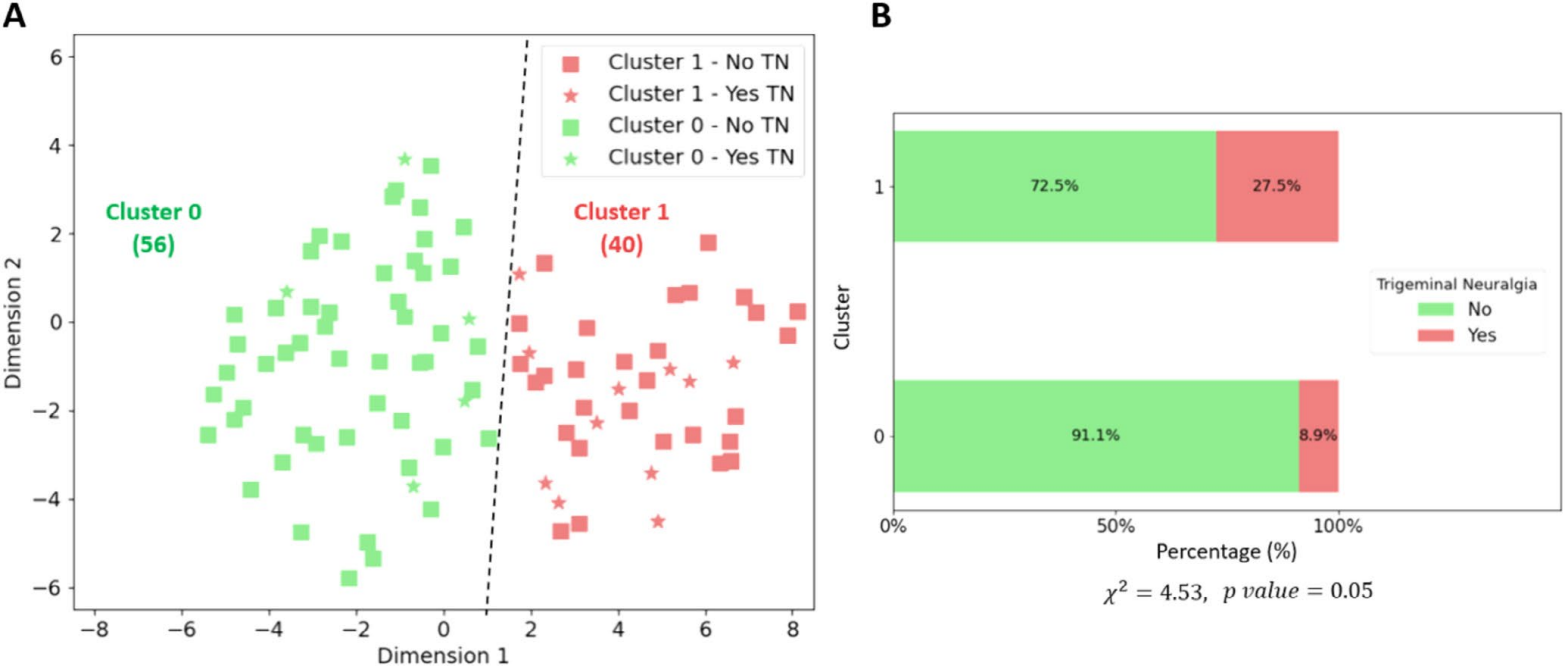
Cluster distribution and association with trigeminal neuralgia. (A) This figure, created with a python code, illustrates the outcome of the unsupervised clustering process conducted using the K‐Means algorithm on the data reduced by the t‐SNE components. Two distinct clusters are shown: Cluster 0 (in green) and Cluster 1 (in red). The stars represent individuals with trigeminal neuralgia (TN) within both clusters. It is notable that the distribution of individuals with TN (stars) is predominantly shifted towards Cluster 1, indicating that most individuals with TN are grouped in this cluster. However, those with TN in Cluster 0 represent individuals who suffer from TN but have not significantly altered their masticatory habits. This spatial distribution highlights the potential role of masticatory behavior in distinguishing TN‐affected individuals. (B) Bar plot showing the percentage of individuals with and without trigeminal neuralgia in each cluster. Cluster 1 has a higher percentage of individuals diagnosed with trigeminal neuralgia. The chi‐square test yielded a *χ* ^2^ value of 4.53 with a *p*‐value of 0.05, indicating a marginally significant association between the clusters and the presence of trigeminal neuralgia. (A) Cluster 0 (in green) and Cluster 1 (in red). The stars represent individuals with trigeminal neuralgia (TN) within both clusters, while the squares represent individuals without TN. (B) The green bars represent the percentage of individuals in the cluster without TN, while the pink bars represent the percentage of those with TN.

Using the two‐component variables calculated by the t‐SNE algorithm, the silhouette score was computed to identify potential clusters. Through an analysis of cluster configurations ranging from two to five, we observed that the configuration with the highest silhouette score of 0.48 corresponded to a cluster number of two (Figure 2). Consequently, two distinct groups were identified (labeled as 0 and 1), encompassing 56 and 40 data points respectively. In the bi‐dimensional space rendered by the t‐SNE components, the data points are segregated as depicted in Figure 3A. This spatial representation illustrates the effective partitioning achieved by the combined use of t‐SNE and K‐Means algorithms, underpinning the distinct clustering observed. An interesting observation from our clustering analysis is the distribution of individuals with trigeminal neuralgia (TN) across the two clusters. As depicted in Figure 3A, while the majority of individuals with TN (represented by stars) are grouped in Cluster 1, a subset of individuals with TN is present in Cluster 0. This suggests that although TN is more prevalent in Cluster 1, where chewing patterns are significantly altered, there are individuals in Cluster 0 who, despite having TN, have not significantly modified their masticatory behavior. These individuals may experience TN but maintain relatively normal chewing patterns, potentially reflecting less severe pain or different coping mechanisms.

### 3.2 Comparative Analysis of Chewing Patterns and Trigeminal Neuralgia Prevalence

The detailed outcomes of the analysis are shown in Table 1. The results revealed distinct chewing patterns between the two clusters. Cluster 0 exhibited a pattern where subjects took fewer bites for both food types (23 bites for apple and 94 for carrot) but exerted greater chewing force (measured at 1.32 ± 1.74 mV/s for apple and 1.29 ± 0.96 mV/s for carrot), with statistically significant differences compared to Cluster 1 (*p* < 0.001 for both apple and carrot chewing force). Conversely, Cluster 1 demonstrated a pattern of more bites (50.75 ± 24.63 for apple and 141.5 ± 61.37 for carrot; *p* < 0.001) but with lesser force applied during chewing (0.66 ± 0.74 mV/s for apple and 0.46 ± 0.13 mV/s for carrot; *p* < 0.001). A notable divergence between the clusters was observed in the prevalence of trigeminal neuralgia. As shown in Figure 3B, the bar plot highlights the distribution of individuals with trigeminal neuralgia across the two clusters. Specifically, Cluster 1 exhibits a noticeably higher proportion of affected individuals (27.5%) compared to Cluster 0 (8.9%). This visual distinction underscores the potential correlation between cluster membership and the presence of the condition. The statistical analysis (*χ* ^2^ = 4.53, *p* = 0.05) supports this observation, suggesting that the clustering algorithm may have captured relevant underlying patterns related to the clinical manifestation of trigeminal neuralgia. Cluster 1 exhibited a significantly higher chewing frequency than Cluster 0 for both foods (2.05 ± 1.39 1/s vs. 1.06 ± 1.08 1/s for apple; 2.32 ± 0.81 1/s vs. 1.08 ± 0.70 1/s for carrot; *p* < 0.001). Regarding chewing time, Cluster 1 took longer to chew the apple (31.98 ± 23.51 s vs. 25.63 ± 13.34 s; *p* = 0.02), while Cluster 0 spent more time chewing the carrot (93.95 ± 51.12 s vs. 70.11 ± 32.20 s; *p* < 0.001). This analysis not only highlights the distinct masticatory patterns between the clusters but also underscores the potential link between chewing behavior and the presence of trigeminal neuralgia.

**TABLE 1:** Comparative analysis of chewing patterns and trigeminal neuralgia prevalence between cluster 0 (*n* = 56) and cluster 1 (*n* = 40): Mean (SD), statistic, and FDR corrected *p*‐values for various features.

Features	Cluster 0 *n* = 56	Cluster 1 *n* = 40	Test statistic	*p* (FDR corrected)
	Median ± IQR	Median ± IQR		
Chewing time‐apple (s)	**25.63 ± 13.34**	**31.98 ± 23.51**	**777**	**0.02** *
Number chews‐apple	**22.75 ± 19.63**	**50.75 ± 24.63**	**193**	**0.00** *
Work—apple (mV/s)	**1.32 ± 1.74**	**0.66 ± 0.74**	**1619**	**0.00** *
Work rate—apple (mV)	0.83 ± 0.70	0.99 ± 1.24	967	0.28
Cycle time—apple (s)	0.04 **±** 0.01	0.04 **±** 0.01	1132.5	0.62
Frequency—apple (1/s)	**1.06 ± 1.08**	**2.05 ± 1.39**	**634**	**0.00** *
Chewing time‐carrot (s)	**93.95 ± 51.12**	**70.11 ± 32.20**	**1511**	**0.00** *
Number chews‐carrot	**90.25 ± 40.00**	**141.50 ± 61.37**	**358**	**0.00** *
Work—carrot (mV/s)	**1.29 ± 0.96**	**0.46 ± 0.13**	**2052.5**	**0.00** *
Work rate—carrot (mV)	5.80 ± 11.20	3.63 ± 6.02	1392	0.06
Cycle time—carrot (s)	0.06 ± 0.1	0.06 ± 0.06	1257	0.31
Frequency—carrot (1/s)	**1.08 ± 0.70**	**2.32 ± 0.81**	**206.5**	**0.00** *
Age (y)	30.00 ± 23.25	35.50 ± 26.00	1009	0.38
Sex (%)	Male: 29	Male: 20	0.51	0.39
	Female: 71	Female: 80		
Smoke (%)	No: 79	No: 60	3.05	0.10
	Yes: 21	Yes: 40		
Wear night bytes # (%)	No: 86	No: 77	0.58	0.39
	Yes: 14	Yes: 23		
Wear dentures (%)	No: 84	No: 95	1.83	0.21
	Yes: 16	Yes: 16		
Preferential chewing side # (%)	Sx: 28	Sx: 28	0	0.64
	Dx: 72	Dx: 72		
Dentoskeletal closure class # (%)	0: 57.5	0: 69	1.12	0.44
	1: 7.5	1: 3.5		
	2: 35.0	2: 27.5		
Short lingual frenulum # (%)	No: 42.5	No: 48.3	0.05	0.57
	Yes: 57.5	Yes: 51.7		
Trigeminal neuralgia (%)	**No: 91.1**	**No: 72.5**	**4.53**	**0.05** *
	**Yes: 8.9**	**Yes: 27.5**		
Dental treatments (%)	No: 91.1	No: 87.5	0.05	0.57
	Yes: 8.9	Yes: 12.5		

*Note*: 1 *T*‐test and Mann Whitney test based on Shapiro–Wilk test for data normality and Chi square test is used for categorical features. Statistically significant differences are reported in bold. The variables marked with # were calculated on a smaller sample as they had missing values.

* Stands for *p*‐value < 0.05.

## 4 Discussion

The findings of this study highlight the significant impact of TN1 on masticatory function and underscore the utility of the “Chewing” device as a tool for objectively assessing these complications. While TN1 is known for intense facial pain, its consequences extend beyond sensory symptoms. Masticatory dysfunction plays a critical role in the disease burden, leading to reduced muscle function, altered eating, and nutritional deficits [20].

Our findings revealed two distinct masticatory patterns in the population, represented by two clusters. Cluster 1, with 27.5% of TN1 individuals, showed more cautious chewing behavior, including increased chewing time and number of chews, compared to Cluster 0 (8.9%). Chewing force in Cluster 1 was reduced by approximately 50% for apples and 64% for carrots. This suggests that individuals in Cluster 1 adapted their chewing behavior in response to pain, though not all in this cluster had TN1, nor were those in Cluster 0 entirely free from it. TN1 presence in Cluster 0 may indicate milder pain or effective coping mechanisms allowing normal chewing. This subclustering reveals heterogeneity within the TN1 population, where masticatory dysfunction varies. Patients in Cluster 1 likely experience chronic pain, with significant adaptations affecting daily activities like chewing. Those in Cluster 0 may have milder symptoms or no link between masticatory activity and pain triggers. Recognizing these patterns is crucial for clinicians to identify chronic stages leading to functional impairments. Patients in Cluster 1 may benefit from therapies targeting both pain and masticatory function, while Cluster 0 patients might prioritize pain relief without interventions for chewing behavior. In healthy individuals, masticatory function typically follows a highly coordinated and efficient pattern. Chewing is characterized by rhythmic, bilateral, and symmetrical activation of the masseter and temporalis muscles, leading to stable and repetitive cycles with adequate chewing force [21, 22]. The food bolus is broken down into small enough particles and mixed with saliva, resulting in a cohesive and easily swallowable mass [22]. On average, normal mastication requires approximately 20–100 chewing cycles per food portion, depending on the texture and hardness of the food [23]. Chewing frequency in healthy individuals typically ranges from 1.0 to 1.5 cycles per second (1/s), which corresponds to approximately 60–90 cycles per minute [24]. Frequencies above 2.0 1/s (~120 cycles/min) are considered abnormally fast and are often used in experimental paradigms to simulate stress‐related or compensatory chewing [21, 22]. In our study, participants grouped in Cluster 0 demonstrated values consistent with these physiological norms. Specifically, the median number of chews was 22.75 for apple (vs. 50.75 in Cluster 1) and 90.25 for carrot (vs. 141.5), and the chewing frequency was 1.06 1/s for apple (vs. 2.05 1/s) and 1.08 1/s for carrot (vs. 2.32 1/s). These data suggest that Cluster 0 reflects a functionally normal masticatory profile, while the significant increase in both chew count and frequency observed in Cluster 1 may indicate compensatory adaptations in response to pain or dysfunction. This interpretation is further supported by existing clinical studies that have documented structural and functional impairments in the masticatory system of TN1 patients [9, 10, 12] Mohammed et al. [9] demonstrated, via imaging and electromyography, significant structural and functional impairments in the masseter muscle after stereotactic radiosurgery, including reduced thickness and strength [9]. Similarly, Montano et al. observed a higher risk of masticatory weakness following percutaneous balloon compression [12], and Ichida et al. [10] reported persistent sensory and motor dysfunction after microvascular decompression, with direct consequences on chewing ability. Zhang et al. [11] confirmed these structural abnormalities via MRI, documenting edema and atrophy in masticatory muscles prior to treatment. Our results corroborate and extend these findings by showing that such alterations are functionally measurable even in non‐operated patients during real‐time chewing tasks, with significantly reduced masticatory force and prolonged chewing duration in a specific subgroup (Cluster 1). Unlike most previous works, which focused on post‐treatment evaluation or static measurements, our study introduces an objective, dynamic method to assess masticatory behavior during actual food processing. This provides additional insight into pain‐adaptive motor patterns and suggests that chewing profiles may be useful not only for diagnosis but also for phenotyping patient subgroups based on functional behavior, independent of clinical labels. However, the real‐time functional data provided by the “Chewing” device offer a complementary perspective, linking these structural alterations to observable changes in masticatory behavior. This approach allows for more dynamic monitoring of the patient's condition and could serve as a valuable adjunct to standard clinical evaluations, which often rely heavily on patient‐reported pain levels. Moreover, the “Chewing” device can also identify masticatory dysfunction in non‐TN individuals, suggesting that it may have broader applications in diagnosing or treating other conditions affecting chewing behavior, such as dental or muscular disorders.

While this study provides compelling evidence for the role of masticatory function in TN1 management, there are several limitations to consider. First, the sample size, while sufficient to demonstrate significant differences between groups, may limit the generalizability of the findings. Future studies should aim to replicate these results in larger, more diverse populations to confirm the robustness of the “Chewing” device as a clinical tool and to refine clinical research and follow‐up therapy protocols, offering a novel avenue for both diagnosis and management of TN1. Second, although the device is highly effective at identifying masticatory dysfunction, it does not address other common triggers of TN1 pain, such as tactile stimuli on the face. Integrating the “Chewing” device with other diagnostic tools may provide a more comprehensive evaluation of TN1 and its multifactorial nature. Moreover, since this was an exploratory study, comprehensive clinical data—such as pain intensity, disease duration, psychological profile, and treatment history—were not systematically collected. The lack of these variables may constitute a potential confounding factor, as they could substantially affect masticatory patterns. Future research should include standardized clinical assessments, such as Visual Analog Scales for pain, validated psychological measures (e.g., anxiety and depression scores), and detailed records of therapeutic interventions, in order to better characterize patient profiles. Although this study focused on objective assessments of masticatory function using electromyographic (EMG) analysis, subjective measures of perceived masticatory capacity and functional limitations were not included. Previous research has demonstrated that conditions such as TN1 can be effectively evaluated using EMG alone, as it provides a precise, real‐time, and non‐invasive assessment of muscle activity and dysfunction [25]. Future studies should consider integrating validated tools such as the Diagnostic Criteria for Temporomandibular Disorders (DC/TMD) [26] and the Jaw Functional Limitation Scale (JFLS‐8) [27] to provide a more comprehensive evaluation of both functional and self‐reported outcomes. The JFLS‐8 [21, 22], for instance, could offer insights into patient‐perceived limitations in chewing, oral movements, and esthetic functions, complementing the quantitative data collected through the “Chewing” device.

As a next step, we aim to develop a machine learning diagnostic model that integrates a larger number of variables, taking into account the complete profile of each subject. This diagnostic algorithm, in conjunction with the “Chewing” device, could also be integrated into environments analyzing metabolic digital twins, such as the Personalized Metabolic Avatar (PMA) [28, 29, 30] enabling automatic diagnosis of the pathology and facilitating the development of personalized therapeutic models.

## 5 Conclusions

The “Chewing” device provides a novel, quantitative, and non‐invasive method to monitor masticatory function in TN patients, offering significant clinical benefits. By identifying distinct masticatory patterns, it enables more targeted treatments, improving outcomes and quality of life. Incorporating such functional assessments into treatment plans allows a more comprehensive approach, addressing both acute pain and long‐term masticatory health. Further research is needed to explore its broader applications and long‐term impact on treatment outcomes in TN and non‐TN populations.

## Author Contributions

Conceptualization: G.M. and A.R. Methodology: G.M., A.R., A.A., G.B., C.S., M.M.D.G., and M.D.S. Software: A.R. and G.M. Validation: G.M., A.R., A.A., G.B., M.M.D.G., and C.S. Formal analysis: G.M. and A.R. Investigation: G.M., A.R., A.A., G.B., C.S, M.M.D.G., M.D.S., M.S., G.C.P., S.C., and R.E. Resources: G.M. and A.R. Data curation: A.R. and G.M. Writing – original draft preparation: G.M. and A.R. Writing – review and editing: A.R., and G.M. Visualization: A.R. and G.M. Supervision: G.M. Project administration: G.M. Funding acquisition: G.M. All authors have read and agreed to the published version of the manuscript.

## Disclosure

Permission to reproduce material from other sources: Reproduction of material requires prior permission.

## Ethics Statement

The study was conducted in accordance with the Declaration of Helsinki and approved by the Ethics Committee of Università Cattolica del Sacro Cuore‐ ID 3147.

## Consent

Informed consent was obtained from all subjects involved in the study. Written informed consent was obtained from the participants to publish this paper. All named authors take full responsibility for all aspects of the work described and we give consent for the work to be published.

## Conflicts of Interest

The authors declare no conflicts of interest.

## Peer Review

The peer review history for this article is available at https://www.webofscience.com/api/gateway/wos/peer‐review/10.1111/jop.70035.

## Supporting information


**Data S1:** Supporting Information.

## Data Availability

The data presented in this study are available on request from the corresponding author. The python code developed in this study are available on request from the corresponding author.
