# Supplementary material for: Using Quantitative Masticatory Dysfunction to Inform Pain Management in Trigeminal Neuralgia Through Electromyographic Monitoring
Source: J Oral Pathol Med. 2025 Aug 15;54(9):863–71. doi: 10.1111/jop.70035 (PMC12521066; doi:10.1111/jop.70035)
Supplement: Supplementary file 1 — Data S1: Supporting Information. [file JOP-54-863-s001.docx]

**Using Quantitative Masticatory Dysfunction to Inform Pain Management in Trigeminal Neuralgia through Electromyographic Monitoring**

Alessia Riente^a, b^, Alessio Abeltino^a, b^, Cassandra Serantoni^a, b^, Michele Maria De Giulio^a, b^, Giada Bianchetti ^a, b^, Mariaconsiglia Santantonio^c^, Giulio Cesare Passali^d^, Stefano Capezzone ^e^, Rosita Esposito^f^, Marco De Spirito ^a, b^ and Giuseppe Maulucci^a, b*^

^a^ Metabolic Intelligence Lab, Department of Neuroscience, Università Cattolica del Sacro Cuore, Largo Francesco Vito, 1, 00168 Rome, Italy; alessia.riente@unicatt.it (A.R., ORCID: 0009-0005-5299-7660); alessio.abeltino@unicatt.it (A.A., ORCID: 0000-0002-8966-5425); cassandra.serantoni@unicatt.it (C.S., ORCID: 0000-0001-7306-8004); michelemaria.degiulio@unicatt.it (M.M.D.G. ORCID: 0009-0004-7712-7576), giada.bianchetti@unicatt.it (G.B., ORCID: 0000-0003-1257-2295); marco.despirito@unicatt.it (M.D.S., ORCID: :0000-0003-4260-5107), [giuseppe.maulucci@unicatt.it](mailto:giuseppe.maulucci@unicatt.it) (G.M., ORCID:0000-0002-2154-319X)

^b^ Department UOC Fisica per le Scienze della Vita, Fondazione Policlinico Universitario “A. Gemelli” IRCCS, 00168 Rome, Italy;

^c^ Complex Operational Unit of Otolaryngology, Bambino Gesù Children’s Hospital, via Della Torre di Palidoro s.n.c., 00050, Fiumicino (Roma), mariaconsiglia.santantonio@opbg.net (M.S., ORCID: 0000-0002-4600-6470);

^d^ Complex Operational Unit of Ear, Nose and Throat Science, Fondazione Policlinico Universitario “A. Gemelli” IRCCS, 00168 Rome, Italy; giuliocesare.passali@unicatt.it (G.C.P., ORCID: 0000-0002-8176-0962);

^e^ Gruppo Fastal Blu Sistemi, via Nomentana 263, 00161 Rome, Italy; stefano@capezzone.it (S.C. ORCID: 0009-0001-1857-5043)

^f^ Digital Innovation Hub Roma, Chirale S.r.l., via Ignazio Persico 32-46, 00154 Rome, Italy; rosita.esposito@chirale.it (R.E.)

*Corresponding Author. Correspondence: giuseppe.maulucci@unicatt.it (G.M. ORCID:0000-0002-2154-319X, tel: 3205730050)

**S2. Materials and Methods**

*S2.1 Data Collection*

Participants were enrolled voluntarly at the Università Cattolica del Sacro Cuore (Rome, Italy) in October 2023. All individuals aged 18 years or older were eligible to participate. After providing informed consent, each participant completed a standardized questionnaire designed to gather demographic information, general health status, and medical history. A structured clinical assessment was then conducted by qualified medical personnel, which included a direct medical examination to evaluate for signs and symptoms of classical trigeminal neuralgia (TN1). For participants who self-reported symptoms consistent with classical trigeminal neuralgia, the diagnosis was confirmed through a brief clinical assessment conducted during the experimental session, in accordance with ICHD-3 diagnostic criteria^1^. Participants who met the diagnostic criteria and reported current TN1 symptoms were classified as TN1 subjects. Those who did not report any history or symptoms consistent with TN1 were categorized as healthy controls. The final dataset therefore included both TN1 patients and healthy participants, allowing for comparative analysis.

The dataset utilized for the analysis comprises data from 96 individuals, enriched with 22 descriptive features. This dataset not only includes details on the chewing patterns of participants for both food types, analyzed using a specialized Python code, but also encompasses a wide array of additional information. The collected data span several categories:

1. Anthropometric Data: This includes age (in years), sex (0=Male, 1=Female), and smoking habits (0=No, 1=Yes).

2. Health and Medical History: Information regarding existing pathologies like classical neuralgia (0=No, 1=Yes), the presence of a short lingual frenulum (0=No, 1=Yes), and dental treatments (0=No, 1=Yes) is gathered.

3. Masticatory Habits: Data on habits such as nocturnal teeth wearing (0=No, 1=Yes), use of dental prostheses (0=No, 1=Yes), preferred chewing side (0=No, 1=Yes), dentoskeletal closure class (class 0,1,2).

A total of 136 individuals were initially screened for eligibility. Forty participants were excluded based on predefined exclusion criteria, including: age under 18, significant facial trauma or surgery, recent radiation therapy, use of complete removable dentures, temporomandibular disorders (TMD), observable bite abnormalities (e.g., such as crossbite, open bite, deep bite, or significant midline deviation), or extensive dental restorations. Specifically, bite abnormalities considered exclusion criteria included crossbite, open bite, deep bite, and significant midline deviation, as these conditions are known to influence masticatory function independently of neuralgic pain. The final selection process is illustrated in Figure S1.


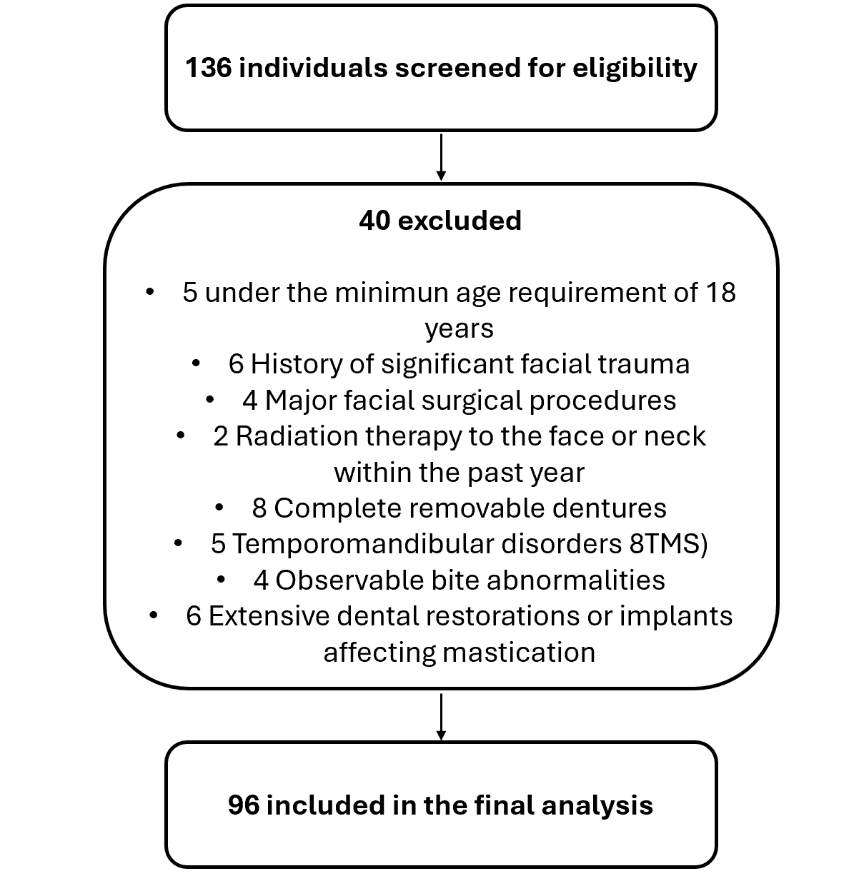


**Figure S1. Flowchart of participant selection process.**

This comprehensive approach allows for a nuanced understanding of the relationship between masticatory behavior and overall health.

*S2.2 Chewing Device*

The evaluation of masticatory behavior in this study is conducted using the 'Chewing' device, an apparatus employing electromyographic technology^2,3^ . While retaining the electronic components of its predecessor, the device has been enhanced with a rechargeable 11V battery, replacing the former 9V battery. This upgrade ensures an extended operational lifespan. Additionally, a more efficient and user-friendly casing for the device has been developed. This casing was fabricated using a laser printer and constructed with plexiglass, offering both durability and practicality (refer to Figure 1a). Figure 1b illustrates the placement of electrodes on the masseter muscles of the subject being tested, a critical step in capturing accurate masticatory data. In addition, Figure 1c displays the EMG trace (in mV) over time (sec) during a chewing session, providing a visual representation of the masticatory activity. The masseter muscle was selected due to its accessibility and the ease of obtaining measurements, making the procedure more comfortable for participants. While other muscles, such as the temporalis and jaw opener muscles, could offer additional insights, the masseter provides a reliable and practical choice.

In the analysis, each time series of masticatory behavior is characterized by six distinct features: 'Chewing Time (sec)', representing the duration of the chewing process; 'Number of Chews', quantifying the total chews within a session; 'Cycle Time (sec)', measuring the duration of a single chewing cycle; 'Work (mV/sec)', a metric of the electrical activity per second; 'Work Rate (mV)', denoting the intensity of muscle activity; and 'Frequency (1/sec)', a new addition. This final feature, 'Frequency', is calculated as the ratio of the 'Number of Chews' to the 'Chewing Time'. For detailed formulations of these metrics and the preprocessing of the data, readers are referred to ^2^, which offers a comprehensive insight into the analytical processes.

*S2.3 Protocol*

Participants first completed an online questionnaire to provide basic information and voluntarily provided written informed consent, confirming their understanding of the food ingredients to prevent any allergic reactions. In this study, two food samples differing in hardness for ingestion by the subjects were prepared: 30 grams of '*Golden Delicious'* apple and 30 grams of carrot. These samples were consistently sourced from the same supermarket on the morning of each test to ensure freshness and uniformity. The maturity level of each sample was assessed using a refractometer, with the apples registering a Brix scale measurement of 13°Brix and 14° Brix^4^. To prepare the food samples, each item was peeled, portioned, and weighed with a professional scale to an exact weight of 30 grams. Each sample was then stored individually in a specialized container to preserve its freshness and crunchiness, ensuring optimal quality for user testing. During the test, subjects were seated comfortably and instructed to consume the samples in a specific order: initially the softer food (apple) followed by the harder one (carrot). They were advised to avoid speaking or moving excessively during the data recording phase and to signal the completion of chewing with a pre-designated gesture. Subjects were asked to eat naturally, adhering to their habitual chewing patterns, to ensure authentic data representation. The measurement method is executed through the strategic placement of three electrodes on each of the subject's masseter muscles. One electrode is positioned at the center of the muscle to capture the primary muscle activity. Another electrode is placed at the terminal part of the muscle, ensuring comprehensive muscle engagement is recorded. The third electrode is affixed to the bony structure adjacent to the muscle, providing a reference point for the muscle's activity.

*S2.4 Data preprocessing*

Initially, a subset of features for analysis was selected: 'Chewing Time (sec) - Apple', 'Number of Chews - Apple', 'Chewing Time (sec) - Carrot, 'Number of Chews - Carrot', and 'Work (mV/sec) - Carrot'. The decision to focus on these specific features was made because they are not correlated with other variables and are considered primary metrics, as the other features, by definition, encompass these key aspects. To ensure comparability and eliminate unit dependency, the input data representing the chewing patterns were standardized.

*S2.5 t-SNE algorithm*

To effectively manage the complexity of our initial high-dimensional feature set, the t-Distributed Stochastic Neighbor Embedding (t-SNE) algorithm for dimensionality reduction^5^ was implemented. t-SNE is specifically engineered for the visualization of multi-dimensional data in a lower-dimensional space (typically two or three dimensions), while meticulously maintaining the inherent structure of the data. The algorithm's core advantage lies in its ability to preserve the local relationships and similarity structures present in the high-dimensional space, ensuring that points closely aligned in the original dataset remain proximate in the reduced representation. The technical workings of t-SNE involve the computation of two distinct probability distributions. The first distribution is derived from the pairwise similarities of the data points in the original high-dimensional space. This similarity is often measured based on the Gaussian distribution of each point’s neighbors. The second distribution represents analogous similarities but in the reduced, lower-dimensional space, typically employing a Student’s t-distribution to account for the reduced degrees of freedom. The primary objective of t-SNE is to minimize the Kullback-Leibler (KL) divergence between these two distributions. This is achieved through a gradient descent process where the algorithm iteratively adjusts the points in the lower-dimensional space to reduce the divergence. This process ensures that the local structure of the data, particularly the relative distances between closely related points, is accurately mirrored in the reduced space. In this specific application, we opted for a two-component solution, effectively projecting the multi-dimensional data onto a two-dimensional plane. This choice was driven by the need for a balance between computational feasibility and the preservation of the data’s intricate structures. t-SNE was employed to reduce the dimensionality to two components, aiming to capture the most salient features of the data and reveal complex, non-linear relationships that traditional methods (like PCA) might miss, thus facilitating more intuitive visualization and analysis while retaining the essential characteristics of the original high-dimensional dataset

*S2.6 Unsupervised clustering with K-Means*

Upon deriving the two-dimensional components through the t-SNE algorithm, unsupervised clustering using the K-Means algorithm was performed. K-Means is a widely recognized clustering technique that partitions a dataset into 'k' distinct clusters, each characterized by its centroid. The algorithm initiates by arbitrarily selecting initial centroids and then iteratively refines these centroids to optimize the clustering. This iterative process involves assigning each data point to the nearest centroid, recalculating the centroids as the mean of the points assigned to them, and repeating these steps until a stable state of convergence is achieved^6^. To ascertain the optimal number of clusters (k), we employed the Silhouette score, a robust metric for evaluating the efficacy of clustering algorithms like K-Means. The Silhouette score provides a measure for each data point, reflecting how appropriately it fits within its assigned cluster in comparison to neighboring clusters. This score ranges from -1 to 1, where higher values signify well-delineated and distinctly separate clusters. A higher average Silhouette score across the dataset indicates superior clustering quality^7^. K-means was selected for its simplicity and effectiveness in clustering, though exploring alternative techniques may provide further insights.

*S2.7 Statistical Methods*

Apart from the chewing pattern features (12), 10 features were categorized as either continuous or categorical variables to facilitate appropriate statistical comparisons between the two clusters. For continuous variables, the Shapiro-Wilk test to assess their distribution was employed. Depending on whether these variables followed a normal distribution, the T-test (for normally distributed variables) or the Mann-Whitney test (for non-normally distributed variables) is applied. Categorical variables were analyzed using the Chi-square test. All hypothesis tests were two-tailed and considered significant at a p-value <0.05. FDR algorithm was applied to the obtained corrected p-values using the Two-Stage Benjamini-Hochberg (TSBH) procedure. Missing data was handled by excluding incomplete cases on a column-by-column basis during the statistical analysis.

**3. Results**

*S3.1* *Study Participants*

In this study, 96 people were involved in carrying out the test. Specifically, the group is composed of 24 men (Sex=0) and 72 women (Sex=1) of which 28 smokers (Smoke=1) and 68 non-smokers (Smoke=0) in an age range between 18 and 71 years (mean age of 35.8±14.1).

**References**

[1] Olesen J. Headache Classification Committee of the International Headache Society (IHS) The International Classification of Headache Disorders, 3rd edition. *Cephalalgia*. 2018;38(1):1-211. doi:10.1177/03331024177382022

[2] Riente A, Abeltino A, Serantoni C, Bianchetti G, De Spirito M, Capezzone S, et al. Evaluation of the Chewing Pattern through an Electromyographic Device. *Biosensors (Basel)*. 2023;13(7). doi:10.3390/bios13070749

[3] Riente A, Abeltino A, Bianchetti G, Serantoni C, DeSpirito M, Pitocco D, et al. Assessment of the influence of chewing pattern on glucose homeostasis through linear regression model. *Nutrition*. Published online September 2024:112481. doi:10.1016/j.nut.2024.112481

[4] table-Brix-fruits-vegetables. https://cdn.idroponica.it/db_img/pdf/tabella-Brix-frutta-verdura.pdf. Accessed September 6, 2023

[5] Van Der Maaten L, Hinton G. Visualizing Data using t-SNE. *Journal of Machine Learning Research*. 2008;9:2579-2605.

[6] Ahmed M, Seraj R, Islam SMS. The k-means algorithm: A comprehensive survey and performance evaluation. *Electronics (Switzerland)*. 2020;9(8):1-12. doi:10.3390/electronics9081295

[7] Shahapure KR, Nicholas C. Cluster Quality Analysis Using Silhouette Score. *IEEE 7th international conference on data science and advanced analytics (DSAA)*. Published online 2020. https://www.
